# Optimization of Polyaluminum Chloride-Chitosan Flocculant for Treating Pig Biogas Slurry Using the Box–Behnken Response Surface Method

**DOI:** 10.3390/ijerph16060996

**Published:** 2019-03-19

**Authors:** Yu Li, Leigang Li, Reham Yasser Farouk, Yuanyuan Wang

**Affiliations:** 1 Key Laboratory of Agricultural Equipment in Mid-Lower Yangtze River, Ministry of Agriculture and Rural Affairs, Huazhong Agricultural University, Wuhan 430070, China; liyu199508@webmail.hzau.edu.cn (Y.L.); Lileigang@webmail.hzau.edu.cn (L.L.); reham.yasser@agr.cu.edu.eg (R.Y.F.); 2 Department of Agricultural Engineering, Faculty of Agriculture, Cairo University, Giza 12613, Egypt

**Keywords:** flocculation, pig biogas slurry, polyaluminum chloride, chitosan, response surface

## Abstract

Flocculation can remove large amounts of nitrogen and phosphorus from wastewater, and the resulting nitrogen- and phosphorus-rich floc can be used to produce organic fertilizer. For biogas slurries containing high levels of nitrogen and phosphorus, ordinary flocculants can no longer meet the flocculation requirements. In this study, to fully utilize the advantages of the two flocculants and achieve efficient removal rates of nitrogen and phosphorus from a biogas slurry, chitosan (CTS) and polyaluminum chloride (PAC) were used as a composite flocculation agent to flocculate pig biogas slurries. The response surface method was used to study the effect of PAC added (PAC_added_) to the composite flocculant (CF), composite flocculant added (CF_added_) to the biogas slurry and the pH on flocculation performance, and optimize these three parameters. In the tests, when the PAC_added_ was 6.79 g·100 mL^−1^_CF_, the CF_added_ was 20.05 mL·L^−1^ biogas slurry and the pH was 7.50, the flocculation performance was the best, with an absorbance of 0.132 at a wavelength of 420 nm. The total phosphorus (TP) concentration was reduced from 214.10 mg·L^−1^ to 1.38 mg·L^−1^ for a removal rate of 99.4%. The total ammonia nitrogen (TAN) concentration was reduced from 1568.25 mg·L^−1^ to 150.27 mg·L^−1^ for a removal rate of 90.4%. The results showed that the CF could form larger flocs, and had greater adsorption capacity and more stable flocculation performance than ordinary flocculants. Furthermore, the CF could exhibit better chelation, electrical neutralization and bridge adsorption.

## 1. Introduction

There are several methods such as adsorption, precipitation, ultra-filtration, electrodialysis, solvent extraction and ion exchange which can be used for the removal of toxic ions. Among these methods, adsorption is the most preferred method [1,2]. Flocculation is an important water treatment technology [3] and has important applications in solid–liquid separation processes [4]. Flocculation not only removes large amounts of nitrogen and phosphorus from wastewater but also converts heavy metal ions in the wastewater into flocs for removal [5]. Biogas slurry is rich in nitrogen, phosphorus, trace elements, and is a potential high-quality nutrient carrier. However, when biogas slurry is treated by ordinary flocculants, the supernatant still cannot be discharged directly because it typically still contains large amounts of nitrogen and phosphorus [6].

The molecular structure of a material substantially determines its final application performance [7,8]. Chitosan (CTS) is a natural polymer derived from renewable resources [9], and it is an important polymer flocculant for water treatment as a cationic polysaccharide. Compared to traditional chemical flocculants, CTS has the following advantages: low dosage, good turbidity removal, high removal efficiency (for chemical oxygen demand (COD), suspended solids (SS) and metal ions), fast deposition rate [10,11,12], easier sludge treatment and no further pollution. However, CTS is more expensive than traditional chemical flocculants [13]. Polyaluminum chloride (PAC) is a cheaper water-soluble inorganic polymer flocculant (IPF) with properties such as adsorption, coagulation, and precipitation [14,15], so it is widely applied to water treatment (Yang et al., 2014; Ali and Kim 2016). But PAC also has some shortcomings, such as instability and corrosivity.

The combined use of PAC and CTS could enhance the flocculation performance of both [16]. Some researchers have found that the decolorization effect of composite PAC-CTS flocculant is better than the single use of PAC and Al-Ferron method [17]. PAC-CTS flocculation of domestic sewage, increased by 36.5% and 21.5%, in terms of COD and turbidity removal rate, respectively, compared with PAC [18]. PAC-CTS flocculated dye waste water and alloying waste water with a decolorization rate and COD removal rate of 99% [19]. Compared with different flocculation processes (CTS, PAC, PAM, PAC-PAM), PAC-CTS has the best flocculation performance in reducing water turbidity, sulfide and odor [20].

However, regarding composite flocculants, most of the current research is focused on the removal of metal ions, turbidity and COD, but not on the nitrogen and phosphorus. In the paper, the flocculant performance in reducing high concentrations of ammonia nitrogen and phosphorus was studied with PAC and CTS. For biogas slurry treatment, flocculants should not degrade the properties of the solid phase, and the addition should be as low as possible [21]. In this study, these two flocculants were combined to form a composite flocculant (CF) and the flocculation parameters, including the PAC added to the CF, the CF added to biogas slurry, and the pH, were optimized. The Box–Behnken response surface method was used to analyze the variation of absorbance, total phosphorus (TP) and total ammonia nitrogen (TAN) concentration of the biogas slurry supernatant after flocculation.

## 2. Materials and Methods

### 2.1. Biogas Slurry

The biogas slurry used in the experiment came from a pig farm located in Ezhou, Hubei Province, China. It was taken from the middle layer of the slurry after fermentation for a period of time. The composition of the pig biogas slurry was complex, and the color was opaque and brownish black. The main physicochemical properties were shown in Table 1.

### 2.2. Test Equipment and Reagents

The following equipment was used in the experiment: a B-type digital display multihead magnetic constant temperature stirrer (HJ-6B, Chengdongshenglian Instrument Factory, Changzhou, China), UV-visible spectrophotometer (UH5300, Shanghai Lai Rui Scientific Instrument Co., Ltd., Shanghai, China), Smartchem200 Discrete Auto Analyzer (SmartChem200, LICA UNITED TECHNOLOGY LIMITED, Beijing, China), automatic microwave extractor (MARS6 CLASSIC, Electronic Scienceand Technology Co.,Ltd. Nanjing Senxi, Nanjing, China), scanning electron microscope (JSM-6390LV, Japanese electronics co., LTD, Tokyo, Japan), CTS with a viscosity of 50–800 mPa·s and deacetylation degree 80.0%–95% (Sinopharm Chemical Reagent Co., Ltd., Shanghai, China), and PAC with a purity of 98% (Shanghai Macklin Biochemical Co., Ltd., Shanghai, China).

### 2.3. Preparation of the CF

CTS (2 g) and acetic acid (2 mL) were completely dissolved in pure water (96 mL) to prepare a CTS–acetic acid solution with a mass fraction of 2%. Then different amount of PAC was added to the CTS–acetic acid solution for adsorption, as shown in Table 2. This mixture was slowly stirred in a constant temperature water bath at 70 °C for1 h, then it was cooled naturally to obtain the CF.

### 2.4. Experimental Design

For the test, 1 L of pig biogas slurry held in a beaker was used. The pH of the biogas slurry was adjusted as given in Table 2 with hydrochloric acid solution and sodium hydroxide solution. The sample was stirred at medium speed (100 r·min^−1^) for 1 min and then stirred rapidly (300 r·min^−1^) as the corresponding amount of CF was added. After 1 min, the sample was stirred slowly (50 r·min^−1^) for 20 min. After the sample was allowed to stand for 45 min, the supernatant was extracted, and its corresponding index was measured.

### 2.5. Analytical Methods

The absorbance of the supernatant at a wavelength of 420 nm was measured with the UV-visible spectrophotometer. Supernatant (1 mL), hydrogen peroxide (1 mL), and concentrated sulfuric acid (5 mL)f were placed in a digestion tube and digested for 45 min at 220 °C in an automatic microwave extractor. TP reacted with antimony potassium tartrate under acidic conditions to form a pale yellow ammonium phosphomolybdate complex, which was then reacted with the reducing agent ascorbic acid to form molybdenum blue. The absorbance was measured at a wavelength of 880 nm, and the absorbance was converted to the TP concentration with the automatic chemical analyzer. Sodium salicylate, ammonium hypochlorite and TAN react to form a blue substance proportional to the TAN concentration, and sodium nitroprusside was added to increase the degree of color development. The absorbance was measured at a wavelength of 660 nm, and the absorbance was converted to the TAN concentration with the automatic chemical analyzer.

### 2.6. Statistical Analysis

The response surface method (RSM) is a tool for developing, improving, and optimizing the statistical and mathematical techniques of a process. The design principles of the Box–Benhnken central combined experimental design was used to develop a three-factor response surface analysis test program. The PAC_added_, the CF_added_, and the pH of the solution were the main factors (independent variables) and represented by X_1_, X_2_, and X_3_, respectively. The absorbance at the wavelength of 420 nm, TP concentration, and TAN concentration of the biogas slurry after the test were the test results (dependent variables) and represented by Y_1_, Y_2_, and Y_3_, respectively. A modol was built to fit to a quadratic polynomial equation by the least-squares method: Y = A_0_ + ∑A_i_X_i_ + ∑A_ii_X_i_^2^ + ∑A_ij_X_i_X_j_. The statistical significance of the model was examined through an analysis of variance (ANOVA) of the polynomial model with a 95% confidence level, and the residual map was used to verify the goodness of the model fit. Finally, the response surface map was analyzed, and the module in the RSM program (Design-Expert.V8.0.6, Stat-Ease, MN, USA) was used to obtain the optimal combination of factor values that met the response requirements.

## 3. Results and Analysis

### 3.1. Development of the Regression Model Equation

Table 2 lists the statistical combinations of key parameters and the statistics of the test results for the three factors. Table 3 presents the regression coefficients and *p*-value of the response function. According to the RSM test data in Table 2, the regression model is given by the following equations regarding the significance of the coefficients.

For the absorbance:

Y_1_ = +10.37 + 0.03X_2_ − 2.09X_3_ + 0.05X_1_^2^ + 0.13X_3_^2^ (1)

For the TP concentration:

Y_2_ = + 195.42 − 1.68X_2_ − 41.62X_3_ + 0.03X_2_^2^ + 2.60X_3_^2^ (2)

For the TAN concentration:

Y_3_ = − 197.71 + 3.11X_1_X_2_ + 9.82X_1_^2^ + 0.44X_2_^2^ − 20.73X_3_^2^ (3)

### 3.2. Verification Model

#### 3.2.1. Analysis of Variance (ANOVA)

Table 3 presents the ANOVA results for the absorbance, TP concentration, and TAN concentration. The p-values of the three test results (dependent variables) are less than 0.01. This means that the second-order polynomial equations (Equations (1)–(3)) are extremely significant, and the models fit well to the experimental results.

The fits of the models are further checked according to the coefficient of determination R^2^. The ANOVA results indicate that the R^2^ values are about 95.00% for all models: 94.44% for the absorbance model, 95.67% for the TP concentration model, and 95.10% for the TAN concentration model. This shows that the polynomial model is accurate.

At the same time, adj R^2^ should be consistent with the rationality of R^2^. In this study, adj R^2^ (absorbance of 87.28%, TP concentration of 90.10%, TAN concentration of 88.80%) has a high value and is close to the R^2^ value, which ensures that the polynomial model made satisfactory adjustments to the test data:

Y_1_: R^2^ = 94.44%, Adj R^2^ = 87.28% (4)

Y_2_: R^2^ = 95.67%, Adj R^2^ = 90.10% (5)

Y_3_: R^2^ = 95.10%, Adj R^2^ = 88.80% (6)

#### 3.2.2. Normal Analysis

**Figure 1:**
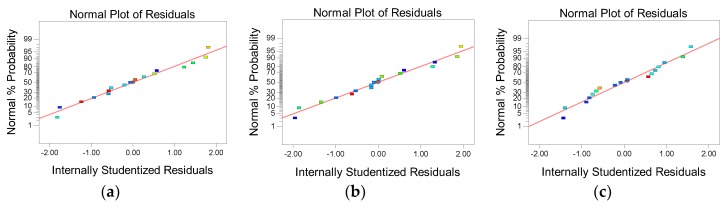
Normal plot of residuals: (**a**) absorbance; (**b**) TP concentration; and (**c**) TAN concentration.

Figure 1 shows the residual and fitted value plots and normal probability plots of the (a) absorbance; (b) TP concentration; and (c) TAN concentration. The normal assumptions for the three test results were relatively satisfactory because the point distributions in the graphs approach a straight line. The residual and predicted graphs were also used to test the fit of the model. In order for the model to be reliable, no outliers should be found. In other words, the points on the graph should be randomly distributed around the zero value on the vertical axis, and there should be no abnormally large or abnormally small residual values. In the figure, the points on the graph are randomly distributed on both sides of the vertical axis, which indicates no problems with the model.

Figure 1 shows that the absorbance, TP concentration, and TAN concentration had a good normal probability distribution and residual performance. Thus, the empirical model can be concluded to be fit for describing the effects of the pH and coagulants used in this study on the absorbance, TP concentration, and TAN concentration as a response surface.

### 3.3. Response Surface Rendering and Parameter Evaluation

#### 3.3.1. Absorbance Response Surface Analysis and Parameter Optimization

There is a certain correlation between the absorbance and the chemical oxygen demand (COD) in the water, which can indirectly reflect the degree of organic pollution [22,23]. Rather than the COD, the absorbance is more feasible and practical that can be used to monitor wastewater. The absorbance value can also reflect the degree of clarification of the water and content of the suspended substance (SS).

The optimization module in the RSM program was used to predict the lowest absorbance. When the PAC_added_ was 6.00 g·100 mL^−1^_CF_, the CF_added_ was 30 mL·L^−1^_biogas slurry_, and the pH was 7.50, the absorbance was 0.102 (the desirability was 1.000).

**Figure 2:**
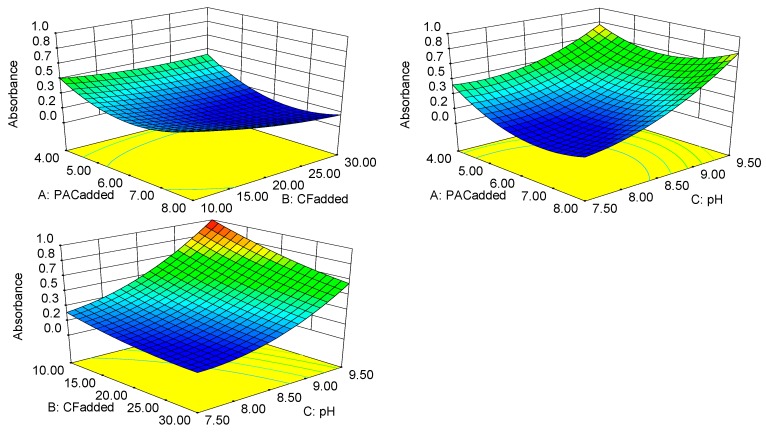
Response surface of the absorbance values.

Figure 2 shows the response surface and contour plots for the PAC_added_, the CF_added_ and pH to absorbance. Response surface plots based on Equation (1) with one variable kept constant at its optimum level, and varying the other two variables within the experimental range. A relatively flat slope for a response surface indicates that the absorbance is robust against variations in the processing conditions. Conversely, if the slope of a response surface is very steep, the response is very sensitive to variation. The shape of the contour line can reflect the strength of the interaction effect. An ellipse indicates that the interaction between two factors is significant, while a circle indicates the opposite. The interaction among the PAC_added_, the CF_added_ and pH was not significant (*p* > 0.05), as shown in Figure 2.

Based on the response surface and contour plots of the absorbance values, the important degree of the three parameters are pH > CF_added_ >PAC_added_. pH could be concluded to be the main factor that affected the absorbance. As the pH increased, the surface became steeper, and the sensitivity of the biogas slurry absorbance to the pH gradually increased. The CF_added_ also affected the absorbance of the biogas slurry. Adding more flocculant lowered the absorbance and improved the treatment effect. Under the optimal conditions, the absorbance was close to zero, which is close to the clarity of pure water. The activity of the CF increased when the pH was closer to neutral, which resulted in better flocculation performance. ANOVA of the model equation showed that the PAC_added_ during the preparation of the CF was not significant and was a secondary factor affecting the absorbance.

CTS was found to dominate the flocculation effect in terms of reducing the absorbance and polyaluminum chloride did not show good flocculation performance. This reflects the strong complexing ability of CTS [24], which can remove colored matter in biogas slurry well. The colored matter in the biogas slurry is mainly suspended in the liquid in the form of particles, and the CF has bridging adsorption. When these particles come into contact with the CF, they are adsorbed onto its long chain. A suitable pH allows the long chain to be more active, which promotes adsorption.

#### 3.3.2. Response Surface Analysis and Optimization of the TP Concentration

The TP concentration is an important index to determine whether or not the biogas slurry can be discharged directly. The optimization module in the RSM program was used to predict the lowest TP concentration. Depending on the difference of the CF_added_ and pH, there was significant variation in the TP concentration after treatment. When the PAC_added_ was 7.8 g·100 mL^−1^_CF_, the CF_added_ was 21.95 mL·L^−1^, and the pH is 7.55, the phosphorus in the biogas slurry was almost completely removed (the desirability was 1.000).

**Figure 3:**
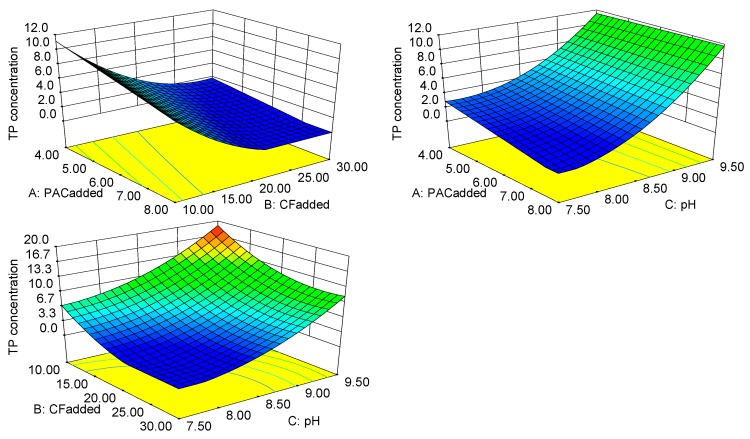
Response surface of the TP concentration.

Figure 3 shows the response surface and contour plots for the PAC_added_, the CF_added_ and pH to the TP concentration. The ANOVA results for the model equation showed that there is no interaction between the three factors, and the PAC_added_ was a secondary factor that affected the removal rate of the TP. But the TP concentration was sensitive to the CF_added_ and pH. Increasing CF_added_ and decreasing pH significantly reduced the TP concentration of the treated biogas slurry. The TP concentration was more sensitive with the variation of CF_added_ and pH when the CF_added_ was above 15.00 mL·L^−1^ and the pH was below 8.65, so greater change of TP concentration was observed in this range, as shown in Figure 3. Compared with other ordinary flocculants, CTS has many advantages, the most obvious of which is the larger flocs that are formed [10,11,12,13]. Larger flocs can better adsorb and carry inorganic substances containing phosphorus in biogas slurry.

The CF made of PAC and CTS can adapt to a wider range of pH [25,26] and reflects a better flocculation performance in a certain pH range. This is because the phosphorus-containing material in the biogas slurry mainly has a negative charge, and the CF is a cationic polymer flocculant. When the CF is contacted with a phosphorus-containing substance, the negatively charged ions carried by the substance undergo a neutralization reaction. This reaction lowers the potential of the phosphorus-containing substance and loses stability. The CF wraps the phosphorus-containing material that loses stability and forms floccules to settle. At the same time, a more neutral pH allows the CF to be more active.

#### 3.3.3. Response Surface Analysis and Optimization of the TAN Concentration

Nitrogen is an abundant component of biogas slurry [27] and an important indicator whether the biogas slurry meets the standards for discharge. The TAN concentration of pig biogas slurry has increased considerably because of changes of pig feed. The unique flocculation characteristics of the CF are reflected in the TAN concentration removal. The optimization module in the RSM program was used to predict the lowest value of the TAN concentration. When the PAC_added_ was 7.13·100 mL^−1^_CF_, the CF_added_ 12.00 mL·L^−1^, and the pH was 7.71, then the TAN concentration was 139.225 mg·L^−1^ (the desirability was 1.000). From the obtained response surface, it was found that the effect of removal TAN in the biogas slurry using the CF was excellent.

**Figure 4:**
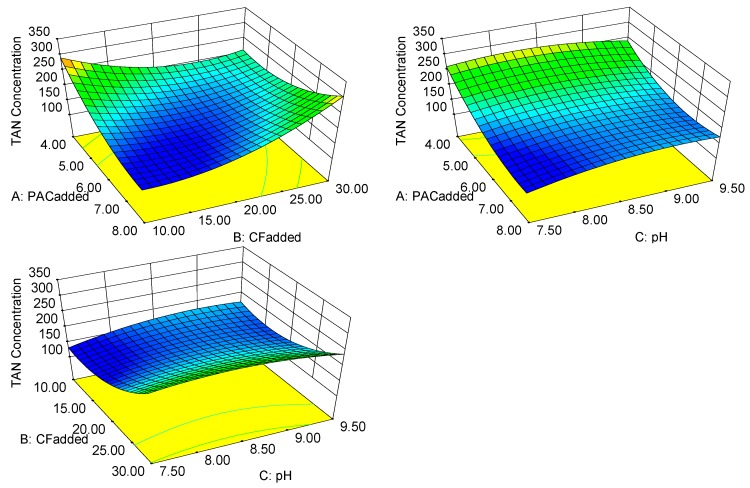
Response surface of the TAN concentration.

According to the ANOVA results for the model equation, all three test factors had insignificant effect on the TAN concentration. Figure 4 shows the response surface and contour plots for the PAC_added_, the CF_added_ and pH to the TAN concentration. The interaction between the PAC_added_ and the CF_added_ was extremely significant (*p* < 0.01), and the other two interactions were not significant (*p* > 0.05). When the PAC_added_ and the CF_added_ were increased or decreased at the same time, the TAN removal from the biogas slurry was affected.

When PAC is complexed with CTS, there is an intermolecular interaction between Al and CTS [27]. An Al–NH_2_ bond is present in the composite flocculant [28,29] that is not conducive to the flocculation of nitrogen-containing organic and inorganic substances in the biogas slurry. This situation led to the mutual inhibition of the removal of TAN after the two flocculants were combined.

PAC_added_ didn’t affect the removal of TAN significantly, but combination of CTS and PAC was concluded to mutually inhibit TAN removal, which may be due to the characteristics of CTS. A large amount of free amino groups are distributed on the CTS molecular chain [30]. CTS also contains a large amount of hydroxyl groups. These hydroxyl groups can form cage-like molecules resembling a network structure by means of hydrogen bonds or salt bonds in the biogas slurry. This molecule chelates with TAN-containing substances in the biogas slurry, so CTS may affect the flocculation of TAN. However, excessive addition of PAC or excessive addition of CTS is not conducive to reducing the concentration of TAN in the biogas slurry. This indicates that the Al–NH_2_ bond is stronger than the chelation reaction.

### 3.4. Optimization of the Flocculation Optimal Conditions

The flocculation test was carried out on pig biogas slurry in order to reduce its absorbance, TP concentration, and TAN concentration. Considered the three measurements as the same weight, the RSM software was used to obtain the minimum absorbance, TP concentration and TAN concentration when Equations (1)–(3) were jointly solved. By consideration three indexes comprehensively, the optimum test conditions were found that the PAC_added_ was 6.79 g·100 mL^−1^_CF_, the CF_added_ was 20.05 mL·L^−1^_biosgas slurry_ and the pH was 7.50. In this case, the biogas slurry absorbance after flocculation treatment was 0.132, the TP concentration was 1.31 mg·L^−1^, the TAN concentration was 145.08 mg·L^−1^, and the desirability was 0.994.

### 3.5. Data Validation and Flocculation Observation

A verification test was carried out under the optimized condition (as shown in Section 3.4). After flocculation treatment, the biogas slurry absorbance was 0.186, the TP concentration was 1.38 mg·L^−1^, the TAN concentration was 150.27 mg·L^−1^. Some scholars added PAC to the biogas slurry first and then added CTS. After the flocculation, the TP removal rate was 75.90%, and the TAN removal rate was 65.50% [31]. Some scholars have used a similar flocculant preparation method, m(PAC): m(CTS) = 4:1, heated in a 70 °C water bath for 1 h, pH 4. The resulting flocculant flocculated a cyanobacterial biogas slurry, and the TP removal rate was 84.05% [32]. In this paper, a composite flocculant was prepared using PAC and CTS firstly, which was used to flocculate biogas slurry later. It was found that the TP removal rate was 99.36% and the TAN removal rate was 90.42% in the biogas slurry. Comparing the two methods, it could be found that CF had good flocculation performance.

**Figure 5:**
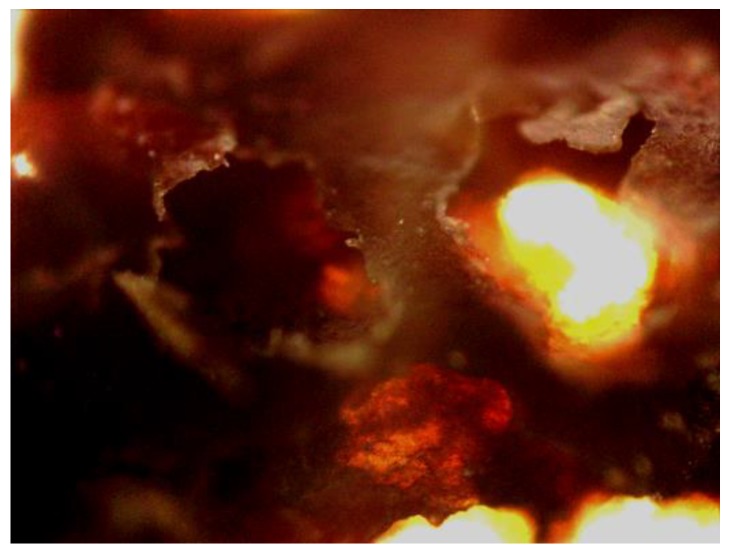
SEM images of floc at 100 magnifications.

The floc was observed by scanning electron microscopy for 100 times magnification, as shown in Figure 5. It was found to be multi-layered and distributed with irregular pores. The contour was obvious and there was no accumulation. This might be because the adhesion had been lost between the flocs after drying.

## 4. Conclusions

In this study, the organic polymer flocculant CTS was modified with acetic acid to form a CTS–acetic acid solution and then combined with the PAC to form a composite flocculant (CF). The amount of flocculant added for flocculation could be reduced, and the cost of CTS could be reduced. The test data were analyzed and organized with RSM software to establish a quadratic mathematical model for the flocculation effect with regard to the absorbance, TP concentration, and total nitrogen concentration. The regression equation could fit the test data well and predict the test results. The response surface of the model and its contour lines were used to explore the key factors and their interactions to optimize the process parameters and improve the flocculation effect. Under the best experimental conditions, the absorbance was 0.132 at a wavelength of 420 nm, the TP removal rate was 99.4% and the TAN removal rate was 90.7%. This composite flocculant used in this study showed improved performance in the reduction of biogas slurry absorbance, TP concentration, and TAN concentration because CF can form larger flocs, and has a greater adsorption capacity and more stable flocculation effect than ordinary flocculants. Furthermore, the CF can exhibit better chelation, electrical neutralization and bridge adsorption. However, the manufacture of the CF has more stringent requirements. For improving flocculation effect, further research should be carried out in later studies on the optimization of flocculant types, in order to improve flocculant quality and reduce cost.

## Figures and Tables

**Table 1:** Physicochemical properties of the biogas slurry for the experiment.

	TAN Concentration (mg·L^−1^)	TP Concentration (mg·L^−1^)	pH
Concentration	1568.25	214.10	8.47

**Table 2:** Box–Behnken design levels and test results.

No.	PAC_added_ (g·100 mL^−1^_CF_)	CF_added_ (mL·L^−1^_biogas slurry_)	pH	Absorbance	TP Concentration (mg·L^−1^)	TAN Concentration (mg·L^−1^)
1	6.00	20.00	8.50	0.459	5.35	160.30
2	8.00	20.00	7.50	0.227	1.39	166.80
3	6.00	20.00	8.50	0.308	5.35	150.51
4	6.00	10.00	7.50	0.176	9.47	158.63
5	6.00	20.00	8.50	0.241	8.48	177.23
6	6.00	30.00	7.50	0.132	1.21	210.90
7	8.00	10.00	8.50	0.808	14.66	177.23
8	4.00	20.00	7.50	0.501	4.63	216.01
9	4.00	10.00	8.50	0.692	18.56	294.46
10	6.00	10.00	9.50	0.972	23.92	190.31
11	8.00	20.00	9.50	0.960	16.44	183.10
12	4.00	20.00	9.50	0.910	15.22	161.80
13	8.00	30.00	8.50	0.325	1.29	324.41
14	6.00	20.00	8.50	0.351	4.13	142.72
15	6.00	20.00	8.50	0.273	5.61	186.20
16	4.00	30.00	8.50	0.400	4.11	192.71
17	6.00	30.00	9.50	0.719	16.51	188.90

**Table 3:** ANOVA results for the absorbance, TP concentration and TAN concentration.

Source	Absorbance			TP Concentration			TAN Concentration		
	Sum of Squares	df	p-Value Prob > F	Sum of Squares	df	p-Value Prob > F	Sum of Squares	df	p-Value Prob > F
Model	1.24	9	0.0013	745.36	9	0.0006	35,484.40	9	0.0008
Residual	0.07	7		33.73	7		1827.56	7	
Lack of fit	0.05	3	0.2464	23.35	3	0.1581	511.94	3	0.6916
Pure error	0.03	4		10.38	4		1315.62	4	
Cor Total	1.32	16		779.09	16		37,311.96	16	

## Abbreviations

**CTS**	chitosan
**PAC**	polyaluminum chloride
**CF**	the composite flocculant
**TP**	total phosphorus
**TAN**	total ammonia nitrogen

## References

[1] Naushad M. (2014). Surfactant assisted nano-composite cation exchanger: development, characterization and applications for the removal of toxic pb2+ from aqueous medium. Chem. Eng. J..

[2] Naushad M., Ahamad T., Sharma G., Al-Muhtaseb H.A., Albadarin A.B., Alam M.M. (2016). Synthesis and characterization of a new starch/sno2 nanocomposite for efficient adsorption of toxic hg2+ metal ion. Chem. Eng. J..

[3] Folens K., Huysman S., Van Hulle S., Du Laing G. (2017). Chemical and economic optimization of the coagulation-flocculation process for silver removal and recovery from industrial wastewater. Sep. Purif. Technol..

[4] Šulc R., Ditl P. (2012). The effect of process conditions on the flocculation process occurring in an agitated vessel. Pol. J. Chem. Technol..

[5] Karbassi A., Marefat A. (2017). The impact of increased oxygen conditions on heavy metal flocculation in the Sefidrud estuary. Mar. Pollut. Bull..

[6] Ali T.U., Kim D.J. (2016). Phosphorus extraction and sludge dissolution by acid and alkali treatments of PAC (PAC) treated wastewater sludge. Bioresour. Technol..

[7] Verma A.K., Dash R.R., Bhunia P. (2012). A review on chemical coagulation·flocculation technologies for removal of colour from textile wastewaters. J. Environ. Manage..

[8] Yang Z., Lu X., Gao B., Wang Y., Yue Q., Chen T. (2014). Fabrication and characterization of poly(ferric chloride)-polyamine flocculant and its application to the decolorization of reactive dyes. J. Mater. Sci..

[9] Kim I.-Y., Seo S.-J., Moon H.-S., Yoo M.-K., Park I.-Y., Kim B.-C., Cho C.-S. (2008). Chitosan and its derivatives for tissue engineering applications. Biotechnol Adv..

[10] Szyguła A., Guibal E., Palacín M.A., Ruiz M., Sastre A.M. (2009). Removal of an anionic dye (Acid Blue 92) by coagulation-flocculation using chitosan. J. Environ. Manage..

[11] Renault F., Sancey B., Badot P.-M., Crini G. (2009). Chitosan for coagulation·flocculation processes – An eco-friendly approach. Eur. Polym. J..

[12] Khawar A., Aslam Z., Javed S., Abbas A. (2018). Pb(II) biosorption using DAP·EDTA-modified biopolymer (chitosan). Chem. Eng. Commun..

[13] Zeng D., Wu J., Kennedy J.F. (2008). Application of a chitosan flocculant to water treatment. Carbohydr. Polym..

[14] Zainol N.A., Aziz H.B.A., Yusoff M.S. (2011). Coagulation and flocculation process of landfill lachate in removing COD, color and ammonia using PAC (PACl). Res. J. Chem. Sci..

[15] Daud Z., Nasir N., Awang H. (2013). Treatment of biodiesel wastewater by coagulation and flocculation using PAC. Aust. J. Basic & Appl. Sci..

[16] Peng T., Li W., Gui-Ying F., Hong-Xia D. (2006). Study on the species distribution and transformation kinetics of pac-cts. J. Northwest Sci-Tech Univ. Agric. For. (Nat. Sci. Ed.).

[17] Zheng W., Migui Z., Lei H., Qiao X., Zizeng L., Lei C. (2017). Research on pac-cts composite flocculant for the decolorization of reactive blue 19. Ind. Water Treatment..

[18] Hongxia M.A., Yaocang L.I. (2012). The preparation and application of chitosan compound flocculant. Meteorol. Environ. Res..

[19] Dawei Y. (2011). Preparation and flocculation of composite flocculant(pac-cts). New Chem. Mater..

[20] Shuyun S., Xiaozhi G.U., Qichao Z., Kaining C. (2016). Research on an emergency treatment technology for black-odor water caused by macrophytes decaying. J. Lake Sci..

[21] Heviánková S., Souček R., Kyncl M., Surovcová N. (2015). Contribution to the study of flocculation of digestate. Geos. Eng..

[22] Huang M.F., Song Q.-J., Mao Z.-H., Xing X.-F., Bai Z.-A., Gu P., Zhao Z.-L. (2011). The retrieval model for COD in waters using optical absorption properties of CDOMa case study at the Shuangtaizi River and the Liaodong Gulf. Acta Oceanol. Sin..

[23] Ferreira-Pinto L., Feihrmann A.C., Tavares C.R.G., dos Reis Coimbra J.S., Saldaña M.D.A., Vedoy D.R.L., Cardozo-Filho L. (2017). Leachate treatment using supercritical water. Can. J. Chem. Eng..

[24] Yang R., Li H., Huang M., Yang H., Li A. (2016). A review on chitosan-based flocculants and their applications in water treatment. Water Res..

[25] Wang Z., Zhong M.-G., Lei H., Xue Q., Lin Z.-Z., Chen L. (2017). Study on flocculation characteristics of humic acid by poly aluminum chloride-chitosan composite flocculant. Environ. Eng..

[26] Şirin S., Trobajo R., Ibanez C., Salvadó J. (2012). Harvesting the microalgae Phaeodactylum tricornutum with PAC, aluminium sulphate, chitosan and alkalinity-induced flocculation. J. Appl. Phycol..

[27] Zhang W.-Y., Zheng Z.-X., Han Y.-F., Zhan M.-F. (2014). Mechanisms of nitrogen and phosphorus adsorption from biogas slurry of piggery by modified zeolite. J. Agro-Environ. Sci..

[28] Yan M., Wang D., Qu J., He W., Chow C.W. (2007). Relative importance of hydrolyzed Al(III) species (Al(a), Al(b), and Al(c)) during coagulation with PAC: A case study with the typical micro-polluted source waters. J. Colloid Interface Sci..

[29] Wang J., Guan J., Santiwong S.R., Waite T.D. (2008). Characterization of floc size and structure under different monomer and polymer coagulants on microfiltration membrane fouling. J. Membr. Sci..

[30] Kumar M.N.V.R., Muzzarelli R.A.A., Muzzarelli C., Sashiwa H., Domb A.J. (2004). Chitosan chemistry and pharmaceutical perspectives. Chem. Rev..

[31] Qingqing C., Jianwei L., Shibing H., Jiaojiao W., Na N., Liqiong W. (2016). Application of PAC-CTS in high concentration pig wastewater. Acta Agric. Boreali-Occident. Sin..

[32] Wenyi Z., Peicheng F., Qiuyan L., Xing L., Mingyuan L. (2012). Polyaluminum chloride-chitosan synthetic composite flocculant and the application in pretreatment of cyanobacteria biogas slurry. Environ. Chem..

